# Effect of selenium-enriched yeast diet on performance, biochemistry, and selenium concentration in meat and egg contents of laying Japanese quails

**DOI:** 10.5194/aab-67-493-2024

**Published:** 2024-10-07

**Authors:** Ziaul Islam, Muhammad Ikram, Shabana Naz, Asad Sultan, Kamran Khan, Ibrahim A. Alhidary, Ruchi Tiwari, Rifat Ullah Khan

**Affiliations:** 1 Department of Animal Sciences, Shaheed Benazir Bhutto University, Sheringal, Dir Upper, Pakistan; 2 Department of Zoology, Government College University, Faisalabad, Pakistan; 3 Department of Poultry Science, Faculty of animal Husbandry and Veterinary Science, The University of Agriculture, Peshawar, Pakistan; 4 Department of Animal Production, College of Food and Agriculture Science, King Saud University, Riyadh, Saudi Arabia; 5 Department of Veterinary Microbiology, College of Veterinary Science and Animal Husbandry, DUVASU, Mathura-281001, UP, India; 6 College of Veterinary Sciences, Faculty of Animal Husbandry and Veterinary Sciences, The University of Agriculture, Peshawar, Pakistan

## Abstract

This study was conducted to determine the effect of a selenium (Se)-enriched yeast-based diet on the performance; blood biochemistry; and Se concentration in the eggs, breast muscle, and some internal organs of laying Japanese quails. A total of 320 healthy female quails were randomly selected and assigned to four dietary groups. The dietary treatment groups consisted of a standard basal diet (control) without supplementation of Se-enriched yeast (SY) and other groups in which Se was supplemented at 1.5 mg (SY-1.5), 2.5 mg (SY-2.5), and 3.5 kg ${}^{-1}$ (SY-3.5). Results showed that quails in SY-3.5 had high ( p<0.05 ) body weight gain. Egg production was improved in SY-2.5 and SY-3.5 groups compared to in the control. Significantly ( p<0.05 ) higher contents of Se in yolk and albumen were observed in the SY-3.5 group. The Se concentration of the breast muscle was higher ( p<0.05 ) in quails of the SY-3.5 group, whereas the liver, kidney, and heart had a high Se content in the SY-2.5 and SY-3.5 groups. Intestinal histological features were improved ( p<0.05 ) in the SY-3.5 group. Overall, this study suggests that adding SY-3.5 to the diet of quails improved their growth and health, as well as the Se content in eggs and meat.

## 1 Introduction

Selenium (Se) is a vital trace mineral that plays a key part in animal production (Zhang et al., 2019; Hassan et al., 2023). Selenium is a major constituent of glutathione peroxidase and other active proteins, e.g., selenoproteins (Dalgaard et al., 2018; Naz et al., 2023). Thus, this mineral species aids different physiological functions of the body, of which the most critically important are oxidoreductase, enhancing fertilization capacity, optimization of embryonic growth, and defense mechanisms (Shakirullah et al., 2017; Zhang et al., 2020; Safiullah et al., 2019). In addition to its antioxidant properties, Se is also involved in other important biological functions, such as immune functioning, thyroid hormone metabolism, and DNA synthesis (Mashaly et al., 2004; Khan et al., 2022; Kang et al., 2022). A deficiency in Se can lead to a range of health problems, including weakened immune functioning, increased risk of certain cancers, and thyroid dysfunction (Qiu et al., 2021). Notably, Se is commonly used in the poultry industry as feed additive to sustain the health and performance of birds (Surai, 2019; Alagawany et al., 2021; Wang et al., 2022). The Se requirement for laying hens is relatively low as an essential mineral element, with a recommended dietary concentration of about 0.3 mg kg ${}^{-1}$ . However, when Se is added to the diet as a nutritional additive, the supplementation level may need to be increased to achieve optimal bioavailability and bio-efficacy (Ohlendorf, 2003).

A number of products are being supplemented in poultry to improve production and health (Gul et al., 2022; Ahmad et al., 2023; Anwar et al., 2023; Maqbool et al., 2023; Rashid et al., 2023; Saad et al., 2023) Nowadays, several Se sources in an organic form (Se-methionine (Se-Met) and Se-enriched yeast) and/or an inorganic form (sodium selenite and nano-Se) are commonly found in poultry feed (Gangadoo et al., 2020). Organic Se sources are generally considered to be less toxic than inorganic sources and can be more readily absorbed and utilized by poultry (Lu et al., 2018, 2020). Moreover, Saccharomyces yeast is commonly used to produce Se-enriched yeast, and it can accumulate 2.85 mg Se g ${}^{-1}$ biomass on a dry-matter basis (Kieliszek et al., 2016). For producing high-quality eggs, in addition to being beneficial for the health of laying hens, the optimum level of Se in the bird's diets is also a nutritional tactic (Abd El-Hack et al., 2017).

In humans, dietary Se could be used to reduce clinical complications brought on by premature birth (Freitas et al., 2014), has therapeutic benefits for people with coronary artery disease (Gharipour et al., 2018), and is crucial for the prevention of the metabolic changes (Damiot et al., 2019). The production of Se-enriched foods is therefore necessary for maintaining human health. Moreover, it is suggested that egg deposition proficiency should primarily be contingent on dietary Se level and Se sources, as well as on the experimental feeding time. Furthermore, there can be a wide dissimilarity in terms of the Se deposition efficacy in eggs among different domestic avian species. Previous studies reported that supplementation in a Japanese quail diet with Se increases the level of Se in eggs (Lukaszewicz et al., 2007). Most studies of egg Se deposition have focused on laying hens that received diets with Se supplementation; quails are rarely investigated in this respect (Zhang et al., 2020). Therefore, this study aimed to investigate the effect of Se supplementation above its basal requirement, using Se yeast as an organic Se, on laying performance, egg quality, egg and tissue Se deposition, and serum biochemistry indices in quails.

### 1.1 Materials and methods

#### 1.1.1 Experimental design and dietary treatments 

In a randomized complete-block arrangement, a total of 320 healthy female quails, aged 20 weeks (average body weight of 221 ± 2.3 g), were randomly distributed into four dietary groups. Furthermore, the quails of each group were split into four replicate groups (20 quails each). The quails of each replicate group were housed in separate cages, reared under a temperature of 23 ± 1 °C and humidity of 65 %–70 % and under a 16L : 8D lighting schedule (where “L” represents light and “D” represents dark). The experimental trial was conducted for 14 weeks, including a 2-week acclimatization period. Each diet (iso-caloric and iso-nitrogenous) was assigned to the four replicate groups. Quails in the control group were fed on a standard basal diet (C) formulated according to their recommended nutritional needs (NRC, 1994; Table 1). The treated groups included basal diets with dietary supplementation with Sel-Plex^®^ (Alltech, USA; containing 0.1 % Se per 500 g) at 1.5 (SY-1.5), 2.5 (SY-2.5), and 3.5 (SY-3.5) mg Se kg ${}^{-1}$ . Moreover, all the birds also had ad libitum availability of clean drinking water.

**Table 1:** Ingredients and composition of the basal diet.

Ingredients	Composition (%)
Soybean meal	30.1
Maize	58.8
Soybean oil	3.3
Dicalcium phosphate	1.2
Salts	0.3
Lime stone	5.7
Vitamin premix ${}^{1}$	0.25
Trace mineral premix ${}^{2}$	0.25
DL-met	0.20
L. Thr	0.2
Estimated nutritional value	
ME (kcal kg ${}^{-1}$ )	2900
Crude protein (%)	22
Crude fiber (%)	2.6
Ether extract (%)	6.2
Phosphorus (%)	0.95
Available phosphorus (%)	0.95
P (analyzed) (%)	0.83
Digestible lysine (%)	1.25
Selenium (mg)	0.15

${}^{1}$ Vitamin premix provides (per kg) vitamin A, 10 000 IU; vitamin D3, 2000 IU; vitamin E, 12 mg; vitamin K3, 1.5 mg; thiamine, 1.5 mg; riboflavin, 4.0 mg; pantothenic acid, 10.0 mg; vitamin B6, 4.0 mg; vitamin B12, 0.02 mg; nicotinic acid, 3.0 mg; folic acid, 0.4 mg; and biotin, 0.1 mg. ${}^{2}$ Mineral premix provides (per kg) Fe or iron and ion carbonate, 60.0 mg; Cu or copper sulfate, 8.0 mg; Mn or manganese oxide, 70.0 mg; Zn or zinc oxide, 50.0 mg; I or calcium iodide, 0.7 mg; and Co or cobalt sulfate, 0.5 mg.

### 1.2 Measurement of growth traits 

Feed intake was recorded daily per replicate. Each individual quail was wing-tagged, and body weight was recorded at the beginning and at the end of the trial. Egg production and egg weight per replicate were noted on a daily basis. Egg weight and quail weight were recorded at a digital-balance authentic accuracy of 0.01 g. For each replicate group, the feed conversion ratio (FCR) was computed (FCR = feed intake/egg mass × 100). Similarly, egg mass per replicate was also determined (egg mass = (egg production (%) × egg weight) × 100) (Nemati et al., 2020).

For determining the egg quality parameters, 24 eggs were randomly chosen from each treatment (six eggs per replicate). The collected eggs were properly kept at normal room temperature until the analysis. All the measurements were taken within 24 h of laying. Each individual egg was weighted using a digital balance. With a digital vernier caliper, the egg length and width (mm) were determined. The eggs were carefully broken, and the egg contents were poured in to the pre-labeled petri plates. For each egg, thick albumen height was determined by means of a digital caliper (0.01 mm). Likewise, the Haugh unit was computed from data on egg weight and albumen height.

Egg shell thickness was measured on its sharp, blunt, and equator end by means of a digital screw gauge. Ratios of egg yolk and albumen shell to egg weight were calculated using the formulas reported by Islam et al. (2021). The yolk, albumen, and shell ratios were calculated as the weight of the yolk, albumen, and shell divided by the weight of the egg, multiplied by 100.

#### 1.2.1 Blood biochemical analysis 

Blood samples were randomly collected from birds of each replicate group (25 % of the group) on the 4th, 8th, and 12th weeks of the trial. The blood ( ∼ 2 mL) was collected from the brachial vein of the quail in an EDTA tube. Blood was then centrifuged at 3500 × g at 4 °C for 10 min to get the serum sample. The serum sample then underwent analysis using a biochemical analyzer to determine the total protein, albumin, glucose, and uric acid (Lu et al., 2019).

### 1.3 Determination of Se in eggs and meat

For determination of Se contents, 10 eggs per replicate (40 eggs per group) were randomly selected on week 4, 8, 12 of the trial and were stored at 25 °C until analysis. The individual egg was marked, weighted, and carefully broken. The yolk and albumen were collected in a chilled tube and then stored at - 30 °C until the analysis. At the end of the 84 d trial, four randomly selected birds from each replicate were slaughtered to collect samples of breast meat and vital organs, including the kidney, liver, and heart. Approximately 10 g of breast meat and 10 g of vital organs were collected from each bird and stored at - 30 °C until analysis. For Se analysis, a 2.0 g aliquot of muscle tissue was harvested, weighed, and homogenized. Samples of 0.5 g from the yolk and breast muscle were taken in a beaker containing concentrated HNO ${}_{3}$ (5 mL) and then were diluted with deionized H ${}_{2}$ O to obtain a 10 mL final volume. The Se analysis was performed using an atomic absorption spectrometer equipped with a graphite furnace module (AAS SOLAAR M6, Unicam, UK) (Muhammad et al., 2021).

#### Histological analysis of intestinal morphology

For intestinal morphology, 4.0 cm segments were taken from each ileum, duodenum, and jejunum of the quails and were processed for histological analysis. The collected segments were fixed in 10 % neutral buffered formalin for 24 h and then were processed for paraffin embedding. Cross-sections of 4 µ m thickness were cut using a microtome (Accu-Cut SRM 200 Sakura) and stained with hematoxylin–eosin dye. Under a light microscope, four vertically oriented villi and their crypts of each group were examined. In each segment, the values of villus height and crypt depth were noted, and the ratio of height to crypt depth was computed for each group.

### 1.4 Statistical analysis

The data analysis for this study evaluating the dietary effect of an Se-enriched yeast diet on the productivity, egg quality, blood biochemical profile, and intestinal morphology of quails was done by means of one-way analysis of variance (ANOVA) using a statistical analysis system (SAS, 1998). To compare the means of each parameter, Duncan's new multiple-range test was used. The level of significance in this case was considered to be p<0.05 .

## 2 Results

### 2.1 Production performance and egg quality parameters

Data showing the effects of an Se-enriched yeast (SY) diet on the production performance of the quails are presented in Table 2. As compared to the control group, weight was significantly ( p<0.05 ) improved in the SY-enriched diet. The highest ( p<0.05 ) body weight was recorded in the SY-3.5 group of the supplemented diet. Similarly, egg production (%) was improved in the SY-enriched diet. High ( p<0.05 ) egg production was reported in the SY-2.5 and SY-3.5 groups. In contrast, feed intake, egg mass, egg weight, and FCR did not vary significantly ( p>0.05 ) among the treatment groups. Data showing the effects of the dietary supplementation of SY on the egg quality parameters are presented in Table 3. Eggshell thickness and Haugh unit value were significantly ( p<0.05 ) affected by Se yeast supplementation. On the other hand, yolk, albumen, and egg shell weight did not differ ( p>0.05 ) as a result of to dietary supplementation of Se-enriched yeast.

**Table 2:** The effect of selenium-enriched yeast supplementation on the productivity of quails.

	Treatments				
Traits	C	SY-1.5	SY-2.5	SY-3.5	p value
Weight gain (g per bird)	19.75 ± 2.59 ${}^{b}$	33.25 ± 3.70 ${}^{\mathrm{ab}}$	33.50 ± 4.97 ${}^{\mathrm{ab}}$	40.75 ± 7.08 ${}^{a}$	0.0214
Egg weight (g)	11.76 ± 0.17	11.83 ± 0.17	12.03 ± 0.18	12.35 ± 0.02	0.2562
Egg production (%)	39.93 ± 0.52 ${}^{b}$	41.437 ± 1.60 ${}^{\mathrm{ab}}$	43.514 ± 1.27 ${}^{a}$	44.65 ± 0.85 ${}^{a}$	0.0433
FI (g d ${}^{-1}$ )	33.88 ± 0.68	34.29 ± 0.85	34.52 ± 0.29	35.03 ± 0.82	0.7050
FCR	3.18 ± 0.28	2.99 ± 0.18	2.98 ± 0.67	2.94 ± 0.62	0.5450
Egg mass	10.83 ± 0.18	11.16 ± 0.88	11.31 ± 0.68	11.59 ± 0.78	0.7350

C – control (basal) diet without supplementation of SY; SY-1.5 – (per kg) basal diet contains 1.5 mg Se; SY-2.5 – (per kg) basal diet contains 2.5 mg Se; SY-3.5 – (per kg) basal diet contains 3.5 mg Se; FI – feed intake; FCR – feed conversion ratio; ns – non-significant ( p>0.05 ). Mean values bearing different superscripts in a row differ significantly from one another ( p<0.05 ).

**Table 3:** Effect of selenium-enriched yeast supplementation on the egg quality of the quails.

	Treatments				
Traits	C	SY-1.5	SY-2.5	SY-3.5	p value
Yolk weight (g)	3.71 ± 0.08	3.62 ± 0.05	3.84 ± 0.04	3.69 ± 0.07	0.148
Yolk weight (%)	31.5 ± 0.50	30.6 ± 0.13	31.2 ± 0.30	30.7 ± 0.36	0.280
Albumen weight (g)	7.29 ± 0.21	7.30 ± 0.22	7.66 ± 0.12	7.45 ± 0.19	0.043
Albumen weight (%)	62.0 ± 1.91	61.7 ± 1.01	62.1 ± 0.31	61.9 ± 0.13	0.996
Shell weight (g)	1.09 ± 0.02	1.08 ± 0.02	1.17 ± 0.04	1.09 ± 0.02	0.868
Shell weight (%)	9.30 ± 0.19	9.13 ± 0.30	9.48 ± 0.20	9.12 ± 0.34	0.756
Shell thickness (mm)	0.23 ± 0.01 ${}^{b}$	0.26 ± 0.88 ${}^{a}$	0.27 ± 0.50 ${}^{a}$	0.27 ± 0.88 ${}^{a}$	0.032
Haugh unit	87.6 ± 0.20 ${}^{b}$	89.4 ± 0.10 ${}^{a}$	88.7 ± 0.30 ${}^{a}$	89.4 ± 0.10 ${}^{a}$	0.001

Means in the same rows bearing different letters (a, b) are significantly different at p<0.05 . C – control (basal) diet without supplementation of SY; SY-1.5 – (per kg) basal diet contains 1.5 mg Se; SY-2.5 – (per kg) basal diet contains 2.5 mg Se; SY-3.5 – (per kg) basal diet contains 3.5 mg Se ( p>0.05 ).

### 2.2 Serum biochemical parameter

Data showing the influence of the supplementation of an SY-enriched diet over the serum biochemical profiles of quails are presented in Table 4. Results show that no significant ( p>0.05 ) differences in the serum biochemical parameters such as glucose, albumin, total protein, and uric acid were observed in quails fed an Se-enriched yeast-based diet.

**Table 4:** Effects of selenium-enriched yeast on serum biochemical parameters of the quails.

		Dietary treatments				
Weeks	Traits	C	SY-1.5	SY-2.5	SY-3.5	p value
4	Glucose (mg dL ${}^{-1}$ )	294.2 ± 0.57	295.5 ± 0.48	295.7 ± 0.60	296.6 ± 0.40	0.071
	Albumin (g dL ${}^{-1}$ )	1.92 ± 0.02	1.94 ± 0.09	1.88 ± 0.016	1.99 ± 0.10	0.785
	Total protein (g dL ${}^{-1}$ )	4.71 ± 0.07	4.79 ± 0.16	4.96 ± 0.07	4.95 ± 0.31	0.077
	Uric acid (g dL ${}^{-1}$ )	4.82 ± 0.27	5.04 ± 0.10	4.93 ± 0.03	4.84 ± 0.07	0.729
8	Glucose (mg dL ${}^{-1}$ )	291.1 ± 0.27	290.4 ± 0.28	293.6 ± 0.80	295.6 ± 0.60	0.081
	Albumin (g dL ${}^{-1}$ )	1.82 ± 0.01	1.84 ± 0.02	1.89 ± 0.012	1.99 ± 0.14	0.685
	Total protein (g dL ${}^{-1}$ )	4.81 ± 0.07	4.89 ± 0.16	4.86 ± 0.07	4.85 ± 0.31	0.067
	Uric acid (g dL ${}^{-1}$ )	4.72 ± 0.27	4.74 ± 0.10	4.78 ± 0.03	4.74 ± 0.07	0.729
12	Glucose (mg dL ${}^{-1}$ )	293.2 ± 0.57	294.6 ± 0.48	292.7 ± 0.60	295.5 ± 0.40	0.071
	Albumin (g dL ${}^{-1}$ )	1.82 ± 0.02	1.98 ± 0.09	1.84 ± .016	1.89 ± 0.10	0.885
	Total protein (g dL ${}^{-1}$ )	4.71 ± 0.07	4.77 ± 0.16	4.76 ± 0.07	4.85 ± 0.31	0.087
	Uric acid (g dL ${}^{-1}$ )	4.72 ± 0.27	4.74 ± 0.10	4.83 ± 0.03	4.74 ± 0.07	0.723

Means in the same rows bearing no superscript are significantly different at p>0.05 . C – control (basal) diet without supplementation of SY; SY-1.5 – (per kg) basal diet contains 1.5 mg Se; SY-2.5 – (per kg) basal diet contains 2.5 mg Se; SY-3.5 – (per kg) basal diet contains 3.5 mg Se; ns – non-significant.

### 2.3 Egg Se concentration

**Figure 1:**
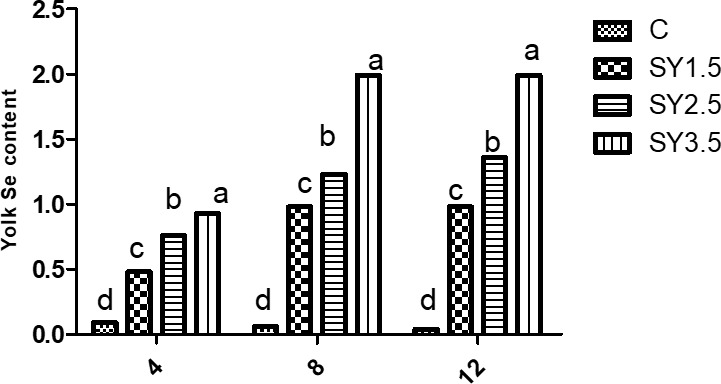
Effect of dietary Se yeast supplementation on the dynamic change in yolk Se contents ( µ g g ${}^{-1}$ yolk) during the 12-week feeding period. C – control (basal) diet without supplementation of SY; SY-1.5 – (per kg) basal diet contains 1.5 mg Se; SY-2.5 – (per kg) basal diet contains 2.5 mg Se; SY-3.5 – (per kg) basal diet contains 3.5 mg Se. Means in the same rows bearing different letters (a, b, c, d) are significantly different at p<0.05 .

**Figure 2:**
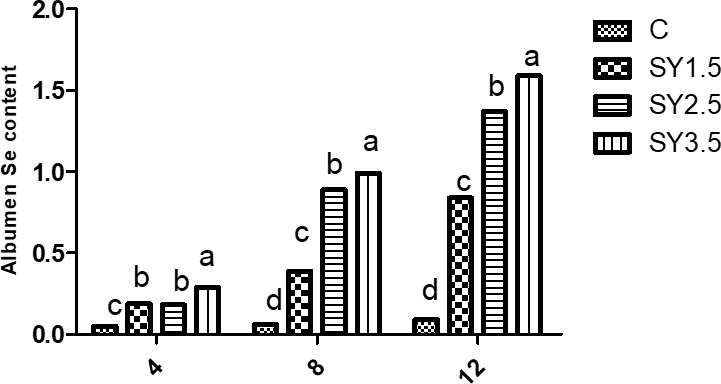
Effect of dietary Se yeast supplementation on the dynamic change in yolk Se contents ( µ g g ${}^{-1}$ albumen) during the 12-week feeding period. C – control (basal) diet without supplementation of SY; SY-1.5 – (per kg) basal diet contains 1.5 mg Se; SY-2.5 – (per kg) basal diet contains 2.5 mg Se; SY-3.5 – (per kg) basal diet contains 3.5 mg Se. Means in the same rows bearing different letters (a, b, c, d) are significantly different at p<0.05 .

Likewise, the Se concentrations of the egg yolk and albumen were significantly ( p<0.05 ) higher in an Se-enriched yeast-based diet than in the control group. Among the supplemented groups, high Se concentrations in egg yolk and albumen were observed in the SY-3.5 group (Figs. 1, 2).

### 2.4 Se concentrations in muscle tissue of quails

**Figure 3:**
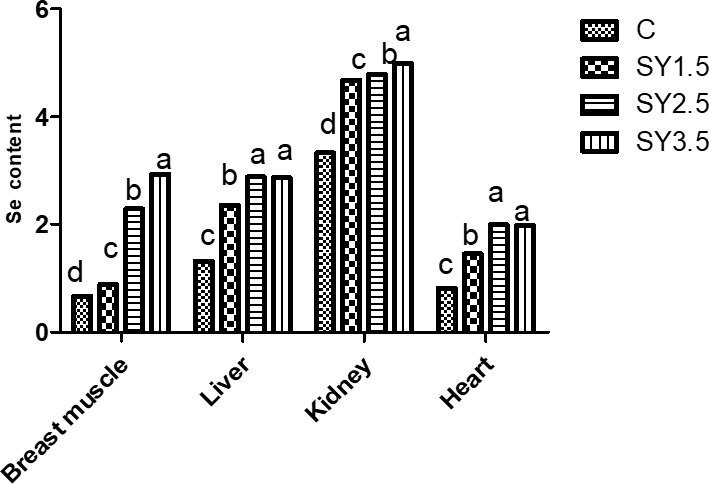
Effect of dietary Se yeast supplementation on the Se contents of the breast muscle and different organs ( µ g g ${}^{-1}$ albumen) during the 12-week feeding period. C – control (basal) diet without supplementation of SY; SY-1.5 – (per kg) basal diet contains 1.5 mg Se; SY-2.5 – (per kg) basal diet contains 2.5 mg Se; SY-3.5 – (per kg) basal diet contains 3.5 mg Se. Means in the same rows bearing different letters (a, b, c, d) are significantly different at p<0.05 .

The Se concentrations of the breast muscle, heart, liver, and kidney as affected by the diet composition are presented in Fig. 3. Supplementation of SY in the quail diet significantly ( p<0.05 ) affected the Se content of the breast muscle, heart, live, and kidney. The Se concentration of the breast muscle was higher in quails of the SY-3.5 group, whereas the liver, kidney, and heart had a high Se content in the SY-2.5 and SY-3.5 groups.

### 2.5 Intestinal morphology of quails

Data on the effects of the SY supplementation on the duodenum, ileum, and jejunum morphology are shown in Table 5. Incorporation of Se-enriched yeast into the quail diet increased ( p<0.05 ) the villus height (VH) and reduced ( p<0.05 ) the crypt depth (CD) in the duodenum, jejunum and ileum. Nevertheless, the VH / CD ratio increased in the supplemented group ( p<0.05 ).

**Table 5:** Effects of selenium-enriched yeast supplementation on the intestinal morphology of quails.

	Dietary group					
Traits		C	SY-1.5	SY-2.5	SY-3.5	p value
Duodenum	VH ( µ m)	866.7 ± 0.45 ${}^{c}$	875.7 ± 0.17 ${}^{c}$	934.2 ± 0.93 ${}^{b}$	984.2 ± 0.34 ${}^{a}$	0.000
	CD ( µ m)	88.3 ± 0.23 ${}^{a}$	86.2 ± 0.50 ${}^{a}$	74.7 ± 0.18 ${}^{b}$	71.3 ± 0.56 ${}^{b}$	0.000
	VCR	9.81 ± 0.03 ${}^{c}$	10.2 ± 0.04 ${}^{b}$	12.5 ± 0.13 ${}^{a}$	13.8 ± 0.12 ${}^{a}$	0.000
Jejunum	VH ( µ m)	742.7 ± 0.11 ${}^{c}$	764.3 ± 0.91 ${}^{b}$	776.3 ± 0.76 ${}^{b}$	786.6 ± 0.31 ${}^{a}$	0.000
	CD ( µ m)	74.5 ± 0.56 ${}^{a}$	73.2 ± 0.65 ${}^{a}$	64.7 ± 0.70 ${}^{b}$	63.8 ± 0.30 ${}^{b}$	0.010
	VCR	9.96 ± 0.01 ${}^{b}$	10.44 ± 0.02 ${}^{b}$	12.02 ± 0.02 ${}^{a}$	11.9 ± 0.02 ${}^{a}$	0.000
Ileum	VH ( µ m)	542 ± 0.52 ${}^{d}$	550.40 ± 0.53 ${}^{c}$	595.67 ± 0.22 ${}^{b}$	620.5 ± 0.40 ${}^{a}$	0.000
	CD ( µ m)	64.57 ± 0.13 ${}^{a}$	66.20 ± 0.57 ${}^{b}$	54.16 ± 0.71 ${}^{b}$	49.5 ± 0.43 ${}^{c}$	0.000
	VCR	8.11 ± 0.12 ${}^{c}$	8.31 ± 0.02 ${}^{c}$	11.0 ± 0.05 ${}^{b}$	12.5 ± 0.04 ${}^{a}$	0.000

Means in the same rows bearing different letters (a, b, c, d) are significantly different significantly at p<0.05 . C – control (basal) diet without supplementation of SY; SY-1.5 – (per kg) basal diet contains 1.5 mg Se; SY-2.5 – (per kg) basal diet contains 2.5 mg Se; SY-3.5 – (per kg) basal diet contains 3.5 mg Se; VH – villus height; CD – crypt depth; VCR – villus height / crypt depth ratio.

## 3 Discussion

Se is an essential trace element that plays a significant part in various biological functions, including immune responses and antioxidant activity (Surai et al., 2018; Ullah et al., 2019). Organic Se supplementation using Se-enriched yeast has become increasingly popular in the poultry industry due to its ability to maintain the birds' health and increase the Se contents in poultry products like meat and eggs. Organic Se, such as selenomethionine (SM), is known to have greater bioavailability and less toxicity than inorganic forms of Se (Markovic et al., 2018). While Se is essential for many physiological processes, excessive intake can lead to toxicity and cause various adverse effects (Kieliszek and Blazejak, 2013). Since Se is an essential nutrient for poultry, it is important to optimize its dietary concentration to ensure good health and performance while avoiding excessive intake that may cause toxicity (Kieliszek and Blazejak, 2016). The use of Se-enriched yeast is considered to be an effective and safe way to boost the Se contents of poultry and their products and to reduce the environmental impact of Se supplementation. Laying hens are often used as a model to study the impacts of different sources and levels of Se on the deposition and bioavailability of this element in eggs and meat (Qiu et al., 2021). Organic Se in the form of Se yeast is generally considered to be safer and more effective than inorganic forms. The dose at which this becomes toxic can vary depending on factors such as the animal species, the form and source of Se, and the duration of exposure. Some studies have reported that high doses of Se yeast can lead to oxidative damage and toxicity in animals, while others have shown no adverse effects (Ullah et al., 2020). Therefore, it is imperative to carefully consider the dose and source of Se when formulating animal diets to ensure that the animals receive optimal nutrition without risking toxicity.

The results of the present study indicated that dietary supplementation of Se had no critical effect on the feed consumption, FCR, egg weight, and mass of Japanese quails. Consistently with results of the present study, Nemati et al. (2020) found that organic-Se sources did not influence theses parameters in laying Japanese quails. The results of the present study indicate that dietary organic Se supplementation significantly enhanced egg production compared with the control group. These results of the present study are confirmed by the findings of some previous researchers. For example, Chitra et al. (2013) reported that egg production was significantly improved by the SY supplementation. Similarly, Han et al. (2017) and Meng et al. (2019) reported that SY supplementation significantly enhanced egg production compared with the control group. The positive response observed in the Se-supplemented groups may have been due to the efficacy of the Se source and its bioavailability. Se plays a vital part in the metabolism and in the action of thyroid hormones in the body that may enhance the egg production (Naylor et al., 2004). The results of this study reveal that the internal egg quality parameters such as egg albumen and yolk weight and their percentages were not affected significantly by the different doses of Se in this study. However, there were slight numerical changes observed over the study period. It should be noted that the egg quality parameters could be influenced by various elements including genetics, diets, health, and optimum environment. Nonetheless, the Se supplementation using different sources in this study tended to have a positive effect on some egg quality traits. Mobaraki and Shahryar (2015) reported similar results following the supplementation of organic Se in quail rations. Se yeast supplementation in the present study positively affected eggshell thickness and Haugh unit, which are important egg quality traits. Zhang et al. (2020) and Baylan et al. (2011) found that supplementation with Se yeast significantly improved eggshell thickness and Haugh unit. Similarly, Nemati et al. (2020) and Maysa et al. (2009) reported that Se-enriched yeast significantly enhanced the eggshell thickness. The improvement in the thickness of eggshells can lead to a reduction in the proportion of broken eggs, which has economic implications for farmers in layer production units (Ketta et al., 2016). The promotion of early fusion in the initial stages of the formation of the shell by means of trace mineral supplementation can improve the mechanical strength of the egg irrespective of shell thickness, as reported by Mabe et al. (2003). Reduced production performance is an external sign of Se toxicity in laying hens, and physiological changes that are related to abnormalities in blood hematology and changes in the histopathology and morphology are considered to be the internal signs (Thiry et al., 2012). Seemingly, these outcomes suggested that 3.5 mg kg ${}^{-1}$ of dietary Se might not cause either Se deficiency or toxicity in the laying hens. It is also reassuring that these results are consistent with earlier findings by Joksimović-Todorović and Davidović (2011). It is important to continuously evaluate and monitor the effects of different levels of Se supplementation to ensure the health and wellbeing of the hens and the quality of the eggs they produce. Se insufficiency could weaken human health, given its association with a weaker immune system and a greater risk of vulnerability to several illnesses (Zhang et al., 2020). The mineral contents of eggs also reflected the nutrition and the health status of laying hens (Attia et al., 2014). Consequently, intake of some functional foods such as egg, meat, and milk enriched with Se is an effective way to improve Se intake (Fisinin et al., 2009). However, the Se content in conventional eggs available in markets is below measurable levels (Khan et al., 2017; Korish and Attia, 2020). Studies confirmed that the supplementation of organic Se into the diets of laying hens translates into better Se content and Se deposition efficiency in their eggs (Tufarelli et al., 2016; Chantiratikul et al., 2018). Since quails may have a better Se transmission rate among poultry, they could be an easier source from which to obtain Se-enriched eggs and meat compared to other species (Nisianakis et al., 2009). Therefore, this study examined the effects of providing an organic selenium source that was above the selenium requirement on egg and meat selenium deposition in laying quails. The results of the present study demonstrate that Se deposition in the eggs and muscle tissue of the quails increased, validating earlier studies of quails (Chantiratikul et al., 2021; Chinrasri et al., 2013). The Se content of eggs from laying quails fed with Se yeast supplementation improved as the feeding days increased. It is notable that the levels of Se transferred to the egg can also be influenced by factors such as the strain of the bird, the concentration and dietary Se sources, and the timing and duration of supplementation (Kim et al., 2018). The supplementation of SY leads to the increased Se concentration in both the eggs and tissues of the hens. It also indicates that the effect was sustained over a period as it was observed on weeks 4, 8, and 12 of the trial. These findings support the practice of using SY as a potential Se source in poultry nutrition for improving the Se content in eggs and tissues. Studies have shown that supplementation in the diets of laying hens with Se-enriched yeast can increase the concentration of Se in both the eggs and tissues of the hens (Lu et al., 2020). The Se concentrations in the eggs and breasts increased as the dietary Se level increased. Pan et al. (2007) noted that Se supplementation improved the Se content of the muscle tissue. Furthermore, quails receiving either organic or inorganic Se appeared to have higher Se concentrations in their kidneys than in other tissues. These findings are consistent with earlier research involving laying quails (Chinrasri et al., 2013). The conversion of ingested Se to H ${}_{2}$ Se and its subsequent use in the synthesis of selenoproteins constitute critical biological processes. However, un-metabolized Se can also accumulate in the body, particularly in the kidneys. This is because the kidneys play a major role in filtering the blood and eliminating waste products, including excess Se. Therefore, long-term exposure to high levels of Se can cause Se toxicity and damage the kidneys (Rayman et al., 2008). Therefore, SY supplementation augmented the Se contents in the albumen, yolk, and breast meat linearly. It seems that the supplementation of SY in the diets of laying Japanese quails had a significant positive effect on egg Se deposition and breast Se concentrations. This study suggested that 3.5 mg kg ${}^{-1}$ of Se enrichment can be safely used in the diet of laying Japanese quails for a period of 84 d without any adverse effects. These findings have useful implication for the consumption of Se-enriched food by humans.

## 4 Conclusions

The findings of this study demonstrated that a selenium-enriched yeast (SY) diet at 3.5 mg kg ${}^{-1}$ effectively improved the productivity of quails. Specifically, quails on this diet exhibited increased body weight gain and enhanced egg production. Moreover, the selenium-enriched yeast led to significantly higher selenium concentrations in the yolk, albumen, and breast muscle, highlighting its effectiveness in enhancing selenium levels in these tissues. This suggests that SY is a potent supplement for boosting selenium content in quail products, which may offer nutritional benefits. Future research should explore and compare the effects of different selenium sources to further understand their bio-efficiency in animal nutrition.

## Data Availability

The data sets used in this paper are available upon request.
